# Phylogeography and Molecular Epidemiology of *Yersinia pestis* in Madagascar

**DOI:** 10.1371/journal.pntd.0001319

**Published:** 2011-09-13

**Authors:** Amy J. Vogler, Fabien Chan, David M. Wagner, Philippe Roumagnac, Judy Lee, Roxanne Nera, Mark Eppinger, Jacques Ravel, Lila Rahalison, Bruno W. Rasoamanana, Stephen M. Beckstrom-Sternberg, Mark Achtman, Suzanne Chanteau, Paul Keim

**Affiliations:** 1 Center for Microbial Genetics and Genomics, Northern Arizona University, Flagstaff, Arizona, United States of America; 2 Institut Pasteur de Madagascar, Antananarivo, Madagascar; 3 Max Planck Institut für Infektionsbiologie, Berlin, Germany; 4 Institute for Genomic Sciences (IGS), School of Medicine, University of Maryland, Baltimore, Maryland, United States of America; 5 Translational Genomics Research Institute, Phoenix, Arizona, United States of America; 6 Environmental Research Institute, University College Cork, Cork, Ireland; Institut Pasteur, France

## Abstract

**Background:**

Plague was introduced to Madagascar in 1898 and continues to be a significant human health problem. It exists mainly in the central highlands, but in the 1990s was reintroduced to the port city of Mahajanga, where it caused extensive human outbreaks. Despite its prevalence, the phylogeography and molecular epidemiology of *Y. pestis* in Madagascar has been difficult to study due to the great genetic similarity among isolates. We examine island-wide geographic-genetic patterns based upon whole-genome discovery of SNPs, SNP genotyping and hypervariable variable-number tandem repeat (VNTR) loci to gain insight into the maintenance and spread of *Y. pestis* in Madagascar.

**Methodology/Principal Findings:**

We analyzed a set of 262 Malagasy isolates using a set of 56 SNPs and a 43-locus multi-locus VNTR analysis (MLVA) system. We then analyzed the geographic distribution of the subclades and identified patterns related to the maintenance and spread of plague in Madagascar. We find relatively high levels of VNTR diversity in addition to several SNP differences. We identify two major groups, Groups I and II, which are subsequently divided into 11 and 4 subclades, respectively. *Y. pestis* appears to be maintained in several geographically separate subpopulations. There is also evidence for multiple long distance transfers of *Y. pestis*, likely human mediated. Such transfers have resulted in the reintroduction and establishment of plague in the port city of Mahajanga, where there is evidence for multiple transfers both from and to the central highlands.

**Conclusions/Significance:**

The maintenance and spread of *Y. pestis* in Madagascar is a dynamic and highly active process that relies on the natural cycle between the primary host, the black rat, and its flea vectors as well as human activity.

## Author Summary

Plague, caused by the bacterium *Yersinia pestis*, has been a problem in Madagascar since it was introduced in 1898. It mainly affects the central highlands, but also has caused several large outbreaks in the port city of Mahajanga, after it was reintroduced there in the 1990s. Despite its prevalence, the genetic diversity and related geographic distribution of different genetic groups of *Y. pestis* in Madagascar has been difficult to study due to the great genetic similarity among isolates. We subtyped a set of Malagasy isolates and identified two major genetic groups that were subsequently divided into 11 and 4 subgroups, respectively. *Y. pestis* appears to be maintained in several geographically separate subpopulations. There is also evidence for multiple long distance transfers of *Y. pestis*, likely human mediated. Such transfers have resulted in the reintroduction and establishment of plague in the port city of Mahajanga where there is evidence for multiple transfers both from and to the central highlands. The maintenance and spread of *Y. pestis* in Madagascar is a dynamic and highly active process that relies on the natural cycle between the primary host, the black rat, and its flea vectors as well as human activity.

## Introduction

Throughout recorded history, *Yersinia pestis*, etiologic agent of plague, has spread multiple times from foci in Central Asia in greatly widening swaths as human-mediated transport became more efficient [1]. Plague attained its current global distribution during the current “third” pandemic, which began in 1855 in the Chinese province of Yünnan, when it was introduced to many previously unaffected countries via infected rats on steam ships [2]. Plague caused widespread outbreaks during this introduction period (∼1900 A.D.), and though disease incidence has since largely decreased, plague remains a significant human health threat due to the severe and often fatal nature of the disease, the many natural plague foci [2] and its potential as a bioterror agent (it is currently classified as a Class A Select Agent [3]). Plague is of particular significance in Madagascar, which has reported some of the highest human plague case numbers (18%–60% of the world total each year between 1995 and 2009) [4] and was the origin of a natural multi-drug resistant strain of *Y. pestis* [5], [6].

Plague has been a problem in Madagascar since its introduction during the current pandemic. It was first introduced to Toamasina in 1898 [7], likely via India [1], with outbreaks in other coastal cities soon after. In 1921, plague reached the capital, Antananarivo, likely via infected rats transported on the railroad linking Toamasina and Antananarivo. Subsequent rat epizootics signaled the establishment of plague in the central highlands [7]. Plague then disappeared from the coast and now exists within two large areas in the central and northern highlands above 800 m in elevation [8]. This elevational distribution of plague is linked to the presence of the flea vectors *Xenopsylla cheopis* and *Synopsyllus fonquerniei*, which are less abundant and absent, respectively, below 800 m [9], [10]. Plague has never disappeared from this region and although it was relatively controlled in the 1950s due to public hygiene improvements and the introduction of antibiotics and insecticides, disease incidence began increasing in 1989 [8], [11], [12]. Human plague cases peaked in 1997 but continue to occur at high frequencies, making Madagascar among the top three countries for human plague cases during the past 15 years [4].

A third, newly emerged plague focus outside the central and northern highlands is the port city of Mahajanga, located ∼400 km by air from Antananarivo [8]. Plague first appeared in Mahajanga during an outbreak in 1902. Subsequent outbreaks occurred in 1907 and between 1924 and 1928 [7]. Plague then disappeared from Mahajanga for a period of 62 years before reappearing during a large outbreak in 1991 [13]. Subsequent outbreaks occurred from 1995–1999 [14]–[16]. During this time, the Mahajanga focus was responsible for ∼30% of the reported human plague cases in Madagascar [14]. Interestingly, this focus likely represents one of the only examples of plague being reintroduced to an area where it had gone extinct, rather than emergence from a silently cycling rodent reservoir without telltale human cases [17].

Molecular subtyping of *Y. pestis* for epidemiological tracking has been difficult due to a lack of genetic diversity [18]. SNP genotyping [1], [19], [20], ribotyping [21], IS*100* insertion element restriction fragment length polymorphism (RFLP) analysis [18], PCR-based IS*100* genotyping [19], [22] and pulsed-field gel electrophoresis (PFGE) [23] have been used to differentiate global isolate collections, however, SNP genotyping provides the most robust phylogenetic reconstructions. SNP genotyping [1], ribotyping [24], IS*100* insertion element RFLP analysis [25], different region (DFR) analysis [26], clustered regularly interspaced short palindromic repeats (CRISPR) analysis [27], ERIC-PCR [28], ERIC-BOX-PCR [28] and PFGE [25], [29] have shown limited to moderate ability in differentiating isolates on a regional scale. Of these, ribotyping has been applied to a set of 187 Malagasy isolates, but only revealed four ribotypes, three of which were unique to Madagascar [24]. SNP genotyping of 82 Malagasy isolates provided greater and more phylogenetically informative resolution, revealing two major groups and an additional 10 subgroups derived from these two major groups that were mostly isolate-specific [1]. In contrast to these other molecular subtyping methods, multi-locus variable-number tandem repeat (VNTR) analysis (MLVA) has shown high discriminatory power at global [19], [30], [31], regional [30], [32]–[35] and local scales [32], indicating its likely usefulness for further differentiation among *Y. pestis* isolates from Madagascar.

The use of SNPs and MLVA together, in a hierarchical approach, has been successfully applied to clonal, recently emerged pathogens [36]–[38]. Point mutations that result in SNPs occur at very low rates, making SNPs relatively rare in the genome, but discoverable through intensive sampling (i.e., whole genome sequencing). In addition, since each SNP likely occurred only once in the evolutionary history of an organism, SNPs represent highly stable phylogenetic markers that can be used for identifying key phylogenetic positions [36]. However, SNPs discovered from a limited number of whole genome sequences will have limited resolving power [36] since they will only be able to identify phylogenetic groups along the evolutionary path(s) linking the sequenced genomes [39]. In contrast, VNTRs possess very high mutation rates and multiple allele states, allowing them to provide a high level of resolution among isolates. Unfortunately, these high mutation rates can lead to mutational saturation and homoplasy which can obscure deeper phylogenetic relationships, leading to inaccurate phylogenies. Using these two marker types together, in a nested hierarchical approach, with SNPs used to identify major genetic groups followed by VNTRs to provide resolution within those groups, allows for both a deeply rooted phylogenetic hypothesis and high resolution discrimination among closely related isolates [36].

We investigated the phylogeography and molecular epidemiology of *Y. pestis* in Madagascar through extensive genotyping and mapping of genetic groups. We genotyped 262 Malagasy isolates from 25 districts from 1939–2005 using 56 SNPs and a 43-marker MLVA system to identify island specific subclades. We then spatially mapped the subclades to examine island-wide geographic-genetic patterns and potential transmission routes.

## Methods

### Ethics Statement

The DNAs analyzed in this study (Table S1) were extracted from *Y. pestis* cultures that were previously isolated by the Malagasy Central Laboratory for plague and Institut Pasteur de Madagascar as part of Madagascar's national plague surveillance plan. The Malagasy Ministry of Health, as part of this national plague surveillance plan, requires declaration of all suspected human plague cases and collection of biological samples from those cases. These biological samples are analyzed by the Malagasy Central Laboratory for plague and Institut Pasteur de Madagascar which also maintains any cultures derived from these samples. These cultures are all de-linked from the patients from whom they originated and analyzed anonymously if used in any research study. Thus, for purposes of this study, all of the DNAs derived from *Y. pestis* cultures from human patients were analyzed anonymously. No Malagasy review board existed during the collection period of the cultures (1939–2001) from which the DNAs used in this study were derived. In addition, the Institutional Review Board of Northern Arizona University, where the DNA genotyping was done, did not require review of the research due to the anonymous nature of the samples.

### DNAs

DNA was obtained from 262 isolates from 25 different districts from 1939–2005 (Figure S1, Table S1). DNAs consisted of simple heat lysis preparations or whole genome amplification (WGA) (QIAGEN, Valencia, CA) products generated from the heat lysis preps. Most of the isolates were collected by the Malagasy Central Laboratory for plague supervised by the Institut Pasteur de Madagascar and were primarily isolated from human cases with a few isolated from other mammals or fleas. A handful of other isolates were from other institutions (still originally collected by the Malagasy Central Laboratory for plague) or represent publically available whole genome sequences (Table S1).

### SNP Genotyping

A total of 56 SNPs were chosen to genotype the Malagasy isolates because they either marked the branches leading to or from the Madagascar clades in a worldwide analysis [1] or were polymorphic among Malagasy isolates (Table S2). These SNPs were either previously identified in a worldwide SNP study on *Y. pestis* using a combination of denaturing High Performance Liquid Chromatography (dHPLC) and whole genome sequence comparisons [1] or identified here through whole genome sequence comparisons among 2 Malagasy whole genome sequences (MG05-1020 [GenBank:AAYS00000000] and IP275 [GenBank:AAOS00000000] [1]) and 14 other *Y. pestis* strain sequences (CO92 [GenBank:AL590842] [40], FV-1 [GenBank:AAUB00000000] [41], CA88-4125 [GenBank:ABCD00000000] [42], Antiqua [GenBank:CP000308], Nepal 516 [GenBank:CP000305] [43], UG05-0454 [GenBank:AAYR00000000] [1], KIM 10 [GenBank:AE009952] [44], F1991016 [GenBank:ABAT00000000], E1979001 [GenBank:AAYV00000000], K1973002 [GenBank:AAYT00000000], B42003004 [GenBank:AAYU00000000] [45], Pestoides F [GenBank:CP000668] [46], Angola [GenBank:CP000901] [20] and 91001 [GenBank:AE017042] [47]). These whole genome sequence comparisons involved comparing the predicted gene sequences of the closed genome of *Y. pestis* strain CO92 [40] to the completed and draft genomes of all other strains using MUMmer and in-house Perl scripts [48]. For genomes with deposited underlying Sanger sequencing read information, a polymorphic site was considered of high quality when its underlying sequence in the query comprised at least three sequencing reads with an average Phred quality score >30 [20], [49].

A TaqMan-minor groove binding (MGB) assay or a melt mismatch amplification mutation assay (Melt-MAMA) was developed for each SNP for use in genotyping the Malagasy DNAs. A TaqMan-MGB assay was designed around one SNP known to divide Malagasy isolates into two major groups (Mad-43, Table S2). Melt-MAMA assays were designed around the other 55 SNPs as previously described [38]. SNP locations, primer sequences, primer concentrations and other information for these assays are presented in Table S2. Primers and probes were designed using Primer Express 3.0 software (Applied Biosystems, Foster City, CA). Each 5 µl TaqMan-MGB reaction contained primers and probes (for concentrations see Table S2), 1× Platinum Quantitative PCR SuperMix-UDG with ROX (Invitrogen, Carlsbad, CA), water and 1 µl of template. Each 5 µl Melt-MAMA reaction contained 1× SYBR Green PCR Master Mix (Applied Biosystems) or 1× EXPRESS SYBR GreenER qPCR Supermix with Premixed ROX (Invitrogen) (for assay-specific master mix see Table S2), derived and ancestral allele-specific MAMA primers, a common reverse primer (for primer concentrations see Table S2), water and 1 µl of diluted DNA template. DNA templates were diluted 1/10 for heat lysis preparations or 1/50 for WGA products. All assays were performed on an Applied Biosystems 7900HT Fast Real-Time PCR System with SDS software v2.3. Thermal cycling conditions for the TaqMan-MGB assay were as follows: 50°C for 2 min, 95°C for 2 min and 50 cycles of 95°C for 15 s and 66°C for 1 min. Thermal cycling conditions for the Melt-MAMA assays were as follows: 50°C for 2 min, 95°C for 10 min and 40 cycles of 95°C for 15 s and 55–65°C for 1 min (see Table S2 for assay-specific annealing temperatures). Melt-MAMA results were interpreted as previously described [38].

### MLVA

All 262 Malagasy isolates were also genotyped using a 43-marker MLVA system as previously described [32].

### Node Assignment

In general, missing SNP data (<0.5% of dataset) were not a factor in node assignment (see SNP phylogenetic analysis below) since data were usually available for an equivalent SNP, thus leading to unambiguous node assignments for most isolates. However, there were four cases where the node assignment was potentially ambiguous. For three isolates missing data for SNP Mad-21 (branch 1.ORI3.k-1.ORI3.o, Table S2), the ancestral allele state was assumed for that SNP for those isolates, since in this and in a previous worldwide analysis [1], only a single isolate, not included among these three, belonged to node “o.” For a single isolate missing data for SNP Mad-46 (branch 1.ORI3.d-1.ORI3.h1, Table S2) the derived state was assumed, due to the placement of that isolate in MLVA subclade II.B in a neighbor-joining analysis and the observed congruence between the “h” nodes and MLVA subclade II.B (see phylogenetic analyses below, Table S1).

### Phylogenetic Analyses

**Figure 1:**
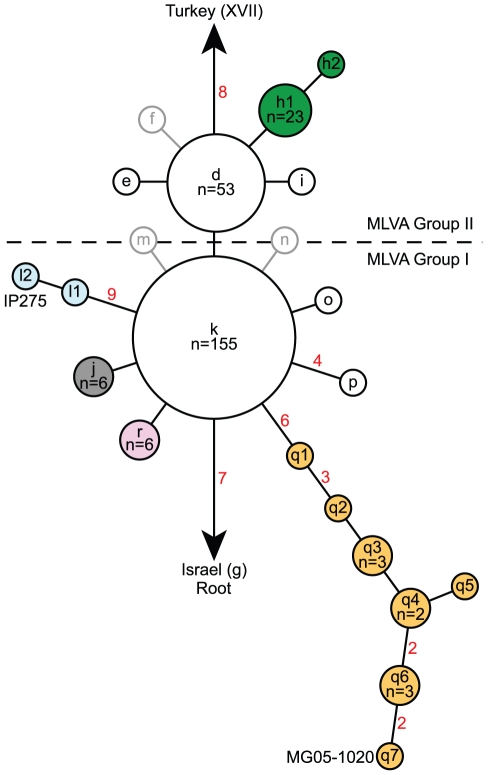
SNP phylogeny of 262 Malagasy isolates. Nodes were named as in Morelli et al. [1] (lower case letters) and belong to the 1.ORI3 group described there [1]. Previously identified nodes [1] that were expanded in this analysis (h, l and q) have additional number designations (e.g., q1) given to each new node in the expansions. The one entirely new node was assigned a new letter, “r.” Previously identified nodes [1] that were not represented by any isolates in this study are represented by gray outlines. Colored nodes correspond to MLVA-identified subclades and are colored the same as their matching MLVA subclades in Figure 2A–B. The number of isolates in nodes with >1 isolate are indicated as are the number of SNPs on branches (red numbers) with >1 SNP. The nodes containing the two sequenced Malagasy strains, MG05-1020 and IP275, are labeled with the strain names.

**Figure 2:**
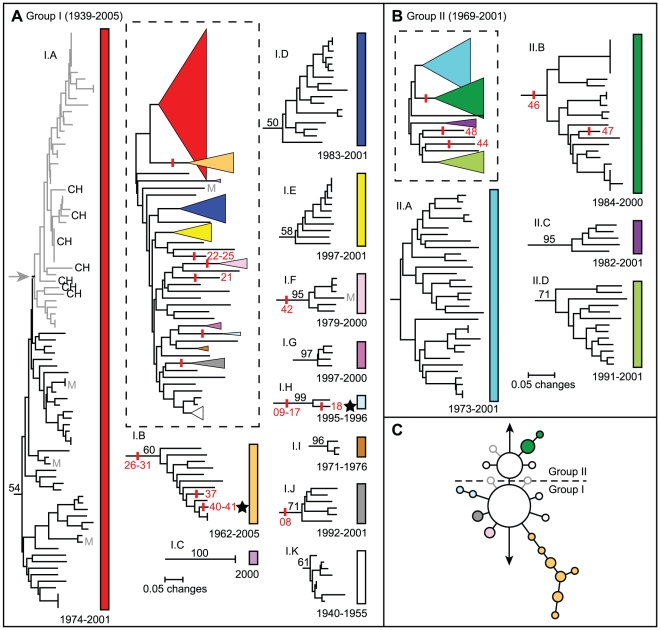
Neighbor-joining dendrograms based upon MLVA data. Dendrograms for Group I (A) and Group II (B) are indicated. The SNP phylogeny from Figure 1 is also indicated (C) for comparison. Subclades within Groups I and II are collapsed in the full phylogenies (dotted boxes) for those groups (colored triangles) and are then individually expanded to show the structure within each subclade. The expanded subclades are labeled based upon their membership in Group I or II and by a capital letter (e.g., I.A) and are indicated by colored bars. Bootstrap values ≥50 supporting individual subclades are indicated on the expanded subclade phylogenies. SNP locations are indicated by vertical red lines. These red lines are labeled with the SNP ID numbers presented in Table S2 on the full phylogenies for unaffiliated isolate-specific SNPs and on the expanded phylogenies for all other SNPs. The years of isolation for isolates within each full and expanded phylogeny are indicated beside the panel label and underneath the individual phylogeny, respectively. The gray subcluster marked by the gray arrow in subclade I.A represents the “Mahajanga I.A subcluster,” a subcluster containing most of the isolates from the Mahajanga plague focus. Seven isolates from the central highlands that also fell within this subcluster are labeled with a “CH.” Five Mahajanga isolates that did not belong in this subcluster are labeled with a gray “M” (A). Black stars indicate the locations of the two sequenced Malagasy strains, MG05-1020 in subclade I.B and IP275 in subclade I.H.

A hierarchical approach was applied to the phylogenetic analysis of the Malagasy isolates. First, a SNP phylogeny was generated using data from all 56 SNPs (Figure 1). Second, neighbor-joining dendrograms based upon MLVA data were constructed using MEGA 3.1 [50] for the two main groups in the SNP phylogeny, Groups I and II (Figure 2A–B). These groups corresponded to the two major Malagasy groups in a previous worldwide analysis [1] and so were separated prior to analyzing with MLVA. The remaining SNPs showing variation among the Malagasy isolates mostly defined subclades observed in the MLVA phylogenies or were specific to single isolates, and so were not used to further separate the isolates prior to applying MLVA. The locations of these additional SNPs are marked on the two MLVA phylogenies where applicable (Figure 2A–B). A small set of SNPs provided very fine-scale resolution of the lineage leading to the whole genome sequenced MG05-1020 strain and are not marked on the MLVA phylogeny due to disagreement between the SNP and MLVA phylogenies on this small scale. Distance matrices for the two MLVA phylogenies were based upon mean character differences. Bootstrap values were based upon 1,000 simulations and were generated using PAUP 4.0b10 (D. Swofford, Sinauer Associates, Inc., Sunderland, MA). Branches with ≥50% bootstrap support and/or supported by one or more SNPs were identified as subclades. One other cluster (II.A) was also considered a subclade despite a lack of bootstrap support because of the proximity of a SNP-defined subclade (Figure 2B).

### Geographic Distribution of Subclades

**Figure 3:**
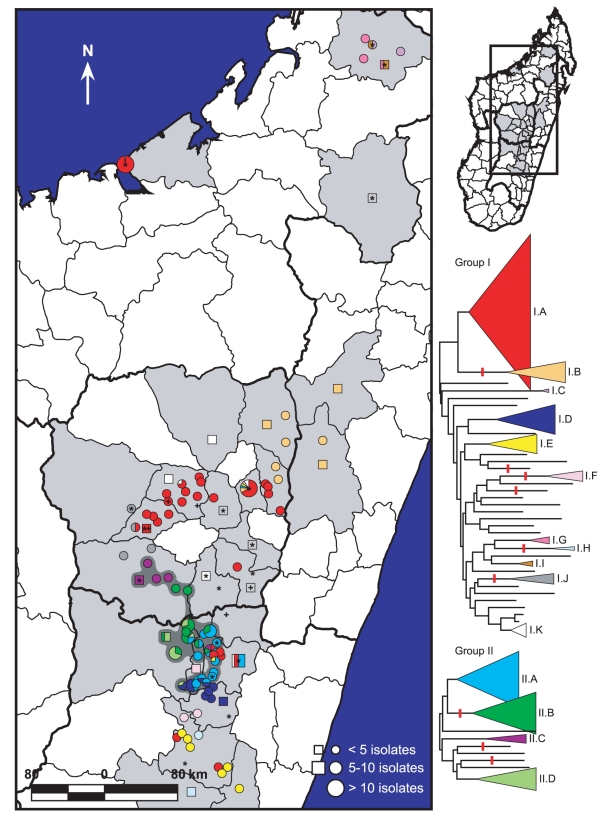
Geographic distribution of MLVA subclades in Madagascar. The MLVA phylogenies for Groups I and II from Figure 2A-B are presented with labeled subclades. Light gray shaded districts indicate Madagascar districts where *Y. pestis* isolates used in this study were obtained. Colors within the mapped circles and squares correspond to the subclade color designations in the MLVA phylogenies. Divisions within those circles and squares indicate that multiple subclades were found at that location. Circles represent isolates where the city/commune of origin is known. Squares represent isolates where only the district of origin is known and are placed within their corresponding districts near to cities/communes containing the same subclade(s) where possible. Six isolates had unknown districts of origin and were not mapped. Unaffiliated Group I and II isolates are indicated by an “*” and a “+,” respectively; these symbols surrounded by a square indicate unaffiliated isolates where only the district of origin is known. The dark gray shaded area indicates the geographic area where Group II subclades are found. Note that some Group I subclades are also found in this area.

We mapped the geographic distributions of the Group I and II subclades we identified to determine their phylogeographic patterns (Figure 3).

### Statistical Analyses

Analysis of similarity (ANOSIM) [51] tests were performed using PRIMER software version 5 to test the hypotheses that 1) Groups I and II form distinct geographic groups and 2) the identified subclades form distinct geographic groups. These tests were performed on all subclades with ≥5 members (N = 221 isolates), thus excluding the unaffiliated isolates and subclades I.C, I.H, I.I and I.G (Table S1). The results of all 55 pairwise comparisons among the subgroups were evaluated at α = 0.000909 (global α of 0.05 divided by 55). To determine if there was a rank relationship between genetic distance and geographic distance, a Spearman correlation coefficient was generated using the RELATE function in PRIMER software with significance of the resulting statistics determined using 10,000 random permutations of the data. This analysis utilized all isolates with any geographic data (N = 256), with district centroids used as the geographic location for isolates for which only district level geographic information was available (N = 33); city/commune point geographic data were used for the remaining 223 isolates. Six isolates lacking any geographic information were excluded from both statistical analyses (Table S1).

## Results

### Genetic Diversity of *Y. pestis* in Madagascar

Our hypervariable-locus and genome-based approaches identified a relatively high level of genetic diversity among the 262 Malagasy isolates from 25 districts from 1939–2005. We confirmed the presence of two major genetic groups, Groups I and II, differentiated by a single SNP, Mad-43 (Figure 1, Table S2), and many VNTR mutational steps. Groups I and II were further differentiated into eleven (I.A–I.K, Figure 2A, Table S1) and four (II.A–II.D, Figure 2B, Table S1) subclades, respectively, based upon MLVA and/or SNPs. All but one of these subclades was at least weakly supported by bootstrap values ≥50 and/or one or more SNPs (Figure 2A–B). The high mutation rates at VNTR loci can lead to homoplasy and, consequently, to low bootstrap support for deeper phylogenetic relationships when analyzing isolates from regional or worldwide collections [19], [34], [36], [52]. Nevertheless, subsequent analyses using more phylogenetically stable molecular markers (i.e., SNPs) have confirmed MLVA-determined clades with weak or even no bootstrap support [19], [38], leading us to use even weak bootstrap support to validate subclades in this analysis. Of the two MLVA identified subclades without bootstrap support, II.A and II.B, subclade II.B was supported by SNP Mad-46 (Table S2) and subclade II.A was designated due to its proximity to and clear separation from the SNP-identified subclade II.B (Figure 2B). Subclades I.B, I.F and I.H were supported by SNPs Mad-26 to 31, Mad-42 and Mad-09 to 17 (Table S2), respectively, and bootstrap analysis (Figure 2A). MLVA also identified 23 and 5 isolates in Groups I and II, respectively, that did not belong to any of the identified subclades within those groups (hereafter referred to as unaffiliated isolates) (Figure 2A–B, I.NONE and II.NONE isolates in Table S1). Four of these unaffiliated isolates and isolates in subclades I.B, I.H and II.B were also identified by apparently isolate-specific SNPs (Figure 2A–B). Overall, MLVA identified 226 genotypes among the 262 isolates, constituting far better resolution than that achieved using ribotyping [24].

The SNP and MLVA analyses showed remarkable congruence. Nearly all of the nodes in the SNP phylogeny either corresponded to MLVA subclades or were specific to individual isolates, allowing the combined analysis of SNP and MLVA data discussed above. Three nodes (f, m and n, Figure 1) did not have representatives in this study, but appeared to be specific for individual isolates in a previous analysis [1]. The only exception to this congruence was within the lineage leading to the whole genome sequenced strain, MG05-1020 (q nodes in Figure 1 and subclade I.B in Figure 2A). In this case, the SNP phylogeny (q nodes, Figure 1) was more accurate than and provided nearly as much resolution as the corresponding MLVA phylogeny (I.B, Figure 2A). This fine-scale phylogenetic resolution was due to the use of a high resolution SNP discovery method, whole genome sequence comparisons, to discover SNPs along this lineage as opposed to the lower resolution dHPLC method used to discover most of the other Malagasy SNPs [1]. Interestingly, comparable resolution was not seen in the lineage leading to the other whole genome sequenced strain, IP275 (l nodes in Figure 1 and subclade I.H in Figure 2A), likely due to the very low number of isolates (N = 2) within that lineage in this analysis.

Missing data for two SNP assays suggested a potential genomic rearrangement (e.g., deletion) in some of the Malagasy strains. Twenty-five of the 262 isolates were missing data for two SNP assays despite repeated attempts at amplification (Table S1). The two SNPs, Mad-28 and Mad-41, were located <850 bp apart at CO92 positions 2,208,345 and 2,207,531, respectively (Table S2), suggesting that there may have been a genomic rearrangement affecting this region in these strains. Intriguingly, IS*100* elements were located flanking these SNPs at CO92 positions 2,135,459-2,137,412 and 2,236,265-2,238,215. IS elements are important facilitators of genomic rearrangements in *Y. pestis* [42], [43] and may have played a role in this result. If so, the same or a similar genomic rearrangement must have occurred multiple times since the 25 isolates were members of six different nodes in the SNP phylogeny (Table S1). This hypothesis is supported by the fact that IS*100* elements are known potential hotspots for genomic rearrangements and excisions in *Y. pestis* [19], [42].

### Geographic Distribution of Isolates

**Figure 4:**
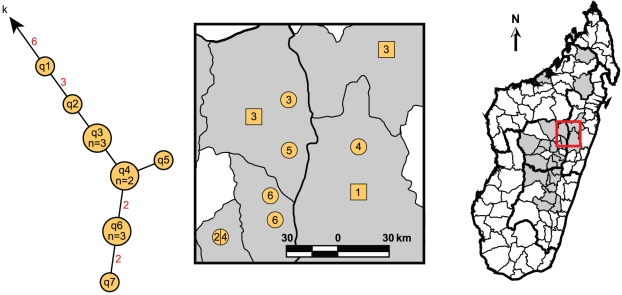
Geographic distribution of SNP-defined nodes in the strain MG05-1020 lineage. The strain MG05-1020 lineage portion of the SNP phylogeny from Figure 1 is indicated as well as an enlarged cutout of the map from Figure 3 showing the geographic distribution of isolates from this lineage. For an explanation of the mapped circles and squares see the figure legend for Figure 3. Circles, squares and pie chart slices in the map are numbered based upon the node number in the SNP phylogeny for the isolates represented by those shapes. The isolate in node “q7” is not mapped due to its geographic origin being unknown.

Significant geographic separation was observed among the identified subclades. Overall, there was a small, but highly significant relationship between genetic and geographic distance (Spearman correlation coefficient ρ = 0.226, *p*<0.0001). In addition, the two main genetic groups, Groups I and II, formed distinct geographic groups based upon an ANOSIM (R = 0.091, *p* = 0.0007). Group II isolates, which possessed the derived state for SNP Mad-43 (Table S2), were essentially restricted to three of the most active plague districts in the central highlands, Betafo, Manandriana and Ambositra [11], and an adjacent district, Ambatofinandrahana (Figure 3, S1). The only exceptions to this were the five unaffiliated Group II isolates, which were scattered in districts to the east and north (+ symbols, Figure 3). In contrast, Group I isolates were found in all three foci, both the central and northern highlands and Mahajanga. Geographic separation among the individual Group I and II subclades was also apparent (Figure 3) and statistically supported in an ANOSIM (R = 0.232, *p*<0.0001). Post-hoc analyses of the pairwise comparisons among subclades indicated that most of the eleven tested subclades formed distinct geographic groups (data not shown). Indeed, several interesting geographic patterns were apparent for the different subclades, only some of which are described below. Separate Group I subclades were found in the northern (I.C, I.G and I.I, Figure 3, Table S1) versus the central (I.A, I.B, I.D, I.E, I.F, I.H, I.J and I.K, Figure 3, Table S1) highlands. Subclade I.A, the largest single subclade, was the dominant subclade found in the capital, Antananarivo, and the surrounding area (Figure 3, S1). With the exception of two isolates, it was also the only subclade found in Mahajanga (Figure 3, S1, Table S1), indicating a central highlands origin for the *Y. pestis* responsible for the series of Mahajanga plague outbreaks from 1991–1999 [13]–[16]. Subclade I.B was the only subclade found in the northeastern portion of the central highlands (Figure 3). Geographic analysis of the corresponding SNP phylogeny (q nodes, Figure 1) for this subclade revealed some additional geographic-genetic patterns. Isolates with the same SNP genotype tended to be clustered geographically, although no distinct spreading pattern could be discerned, possibly due to the limited number of isolates (Figure 4). Subclade I.E was predominantly found in the southern central highlands, in district Fianarantsoa, and also appears to be the subclade responsible for the reemergence of plague in the Ikongo district [53], adjacent to Fianarantsoa on the southeast (Figure 3, S1).

Three subclades, I.F, I.H and I.K, did not show distinct geographic patterns (Figure 3). In the cases of subclades I.F and I.H, this may be due to the limited numbers of isolates within those subclades (Figure 2A, Table S1). The geographically widespread nature of subclade I.K isolates, however, may be related to their older dates of isolation. All of the subclade I.K isolates were isolated between 1940 and 1955 (Figure 2A, Table S1), just 19–34 years after plague was introduced to the central highlands. Therefore, these isolates may represent a subclade that was formerly spread throughout much of the central highlands but that currently does not exist in nature in Madagascar. Similarly, subclade I.I, although it was not geographically widespread (Figure 3), only contained isolates isolated from 1971–1976 (Figure 2A, Table S1) and may represent a former, now extinct subclade from the northern highlands. However, the limited number of isolates makes this difficult to determine. Alternatively, these subclades may still exist, but may have decreased in frequency and/or be very rare in nature.

Interestingly, the other older isolates tended to be the unaffiliated isolates. Eighteen of the 28 unaffiliated isolates were isolated between 1939 and 1978. Another 3 had unknown dates of isolation (Table S1). Given their older dates of isolation, these unaffiliated isolates may also be representatives of older, now extinct subclades from Madagascar. The lack of comparable isolates to these unaffiliated isolates among the rest of the isolate collection could be due to the limited sampling from earlier years (Table S1). Alternatively, the unaffiliated isolates may simply be representatives of very rare subclades. A final possibility could involve the accumulation of VNTR mutations due to repeated passages associated with prolonged storage in the laboratory, which could lead to the older isolates being inaccurate representatives of the original isolates. This is unlikely, however, as the rate of VNTR evolution in the laboratory, even with passaging, should be much slower than in nature. Thus, while these isolates may not be exactly the same as when they were first isolated, they should be close. Also, multiple copies of a subset of the Malagasy isolates in this study that were stored at different temperatures showed identical MLVA genotypes (data not shown), indicating that these VNTR loci are relatively stable in these isolates under the storage conditions used. Regardless, the unaffiliated nature of many of the older isolates is consistent with and most likely related to their older dates of isolation.

Several cities and communes yielded isolates of subclades predominantly found elsewhere, suggesting importation from other locations. Antananarivo, in particular, contained isolates from five subclades in addition to the dominant subclade (Figure 3, S1). Commune Andina Firaisana in the Ambositra district is another example, containing representatives of four different subclades (Figure 3, S1). One of these, subclade I.A, was also found in the nearby surrounding area. However, this area is considerably south of the area where the majority of subclade I.A isolates were found, suggesting that this subclade may have been imported to this area from further north or vice versa (Figure 3). Of the other three subclades found in Andina Firaisana, subclades II.A and II.B are also found in nearby areas and so may be naturally occurring in Andina Firaisana rather than due to transfer events. Subclade II.C, in contrast, appears to have been transferred to Andina Firaisana from the Betafo district in the northwest or vice versa (Figure 3, S1). Another nearby commune, Ivato, contained a single subclade I.E isolate, suggesting a transfer event from district Fianarantsoa in the south (Figure 3, S1).

### Plague in Mahajanga

Our data suggest that *Y. pestis* was reintroduced to Mahajanga from the central highlands. The majority of the Mahajanga isolates (39 of 44) belonged to a single subcluster within subclade I.A (hereafter referred to as the Mahajanga I.A subcluster) (Figure 2A), suggesting that there was an introduction to Mahajanga from the central highlands that became established in Mahajanga and then underwent local cycling. Though this Mahajanga I.A subcluster did not have either SNP or MLVA support (Figure 2A), close examination of the isolates within this subcluster revealed very close genetic relationships, with most differences involving only a single repeat change at a single VNTR locus (data not shown). This is consistent with an outbreak scenario originating from a single introduction and strengthens the identification of this subcluster as a genetic group. In contrast, subclade I.A isolates outside of the Mahajanga I.A subcluster exhibited much greater variation both in the number of VNTR loci displaying polymorphisms and the number of alleles observed at those loci (data not shown), consistent with an older, more geographically dispersed and more differentiated set of isolates.

Our data also suggest that there have been multiple transfers of *Y. pestis* between Mahajanga and the central highlands. Specifically, seven isolates within the Mahajanga I.A subcluster were isolated from central highland locations rather than from Mahajanga (Figure 2A), suggesting that *Y. pestis* was also transferred back from Mahajanga to the central highlands. Two other Mahajanga isolates belonged to subclade I.F and were unaffiliated, respectively (Figure 2A), suggesting that there has been more than one introduction of *Y. pestis* to Mahajanga as well. The final three Mahajanga isolates, although they belonged to subclade I.A, were not part of the Mahajanga I.A subcluster and were instead more closely related to subclade I.A isolates from the central highlands (Figure 2A), again suggesting multiple introductions. However, it is unclear as to whether any of these other introductions became established in Mahajanga due to the lack of other Mahajanga isolates similar to these five outliers. Finally, although our data suggest that there have been multiple transfers of *Y. pestis* between Mahajanga and the central highlands, there is no evidence in these data for an introduction to Mahajanga from the northern highlands, as was previously suggested by PFGE analyses [14], [17].

## Discussion

Madagascar is one of the most active plague regions in the world. However, few studies have investigated the molecular epidemiology of *Y. pestis* from Madagascar and none have done so using very high resolution genomic methodologies. Here, we investigated the phylogeography and molecular epidemiology of *Y. pestis* in Madagascar by using a combination of SNPs and MLVA to analyze 262 Malagasy isolates from 25 districts from 1939–2005. In contrast with previous analyses that utilized ribotyping or SNPs alone [1], [24], we identified a very high level of genetic diversity with 226 MLVA genotypes among the 262 isolates. These genotypes were distributed amongst 15 subclades that displayed significant geographic separation (Figure 3), leading to insights into the maintenance and spread of plague in Madagascar.

The use of MLVA was particularly effective at identifying genetic groups in Madagascar. SNPs, though useful, mostly provided confidence in genetic groups that were already apparent via MLVA. This is somewhat counter to the conventional hierarchical approach wherein SNPs are used first to identify major genetic groups followed by MLVA to provide resolution within those groups, thus minimizing the problems of mutational saturation/homoplasy that can occur with highly variable markers such as VNTRs [36]. In this study, only SNP Mad-43 (Table S2), which differentiated Groups I and II, was useful in this conventional sense to identify “major genetic groups” that were obscured in the MLVA phylogeny (data not shown). All of the other subclades identified by SNPs were also identified by MLVA, suggesting that at this regional scale, MLVA alone may be effective at identifying robust genetic groups. Importantly, though MLVA was excellent at identifying these genetic groups, the relationships among those groups, such as the division between Groups I and II, remained unclear using MLVA alone (data not shown) whereas they were very clearly depicted as a star phylogeny in the SNP phylogeny (Figure 1). Where knowledge of deeper genetic relationships or fine-scale phylogenetic analysis of specific lineages (e.g., the strain MG05-1020 lineage here) is desired, SNPs will remain the preferred methodology for clonal pathogens such as *Y. pestis*. However, until whole genome sequencing for entire isolate collections becomes feasible, MLVA will continue to be a useful tool for examining genetic diversity whether used in conjunction with SNPs or alone.

Our analyses suggest that plague is being maintained in Madagascar in multiple geographically separated subpopulations. We revealed significant geographic separation among the identified subclades (Figure 3), suggesting that these subclades are undergoing local cycling with limited gene flow from other subclades. This is consistent with the population genetics and ecology of the black rat (*Rattus rattus*), the primary plague host in rural Madagascar [7], [9]. The black rat in Madagascar exhibits limited gene flow between subpopulations [54] as well as limited geographic ranges [55]. This limited mobility, a high reproduction rate [10] and the development of some resistance to plague [56] are all likely important factors that allow the black rat to maintain plague in these genetically distinct, geographically separated subpopulations. The two flea vectors, *X. cheopis* and *S. fonquerniei* [9], [10], may also play a role in maintaining genetically distinct subpopulations (i.e., Groups I and II), though more data would be needed to confirm this hypothesis.

In contrast, transport of *Y. pestis* across longer distances in Madagascar is likely human-mediated. Historically, there is ample evidence for the influence of human traffic on the spread of plague, including transport along trade routes such as the Silk Road in the early pandemics and transport via steam ship to numerous new locations during the “third” pandemic [1], [2]. The SNP phylogeny determined by Morelli et al. [1] suggests the progression of plague from Israel to Madagascar to Turkey (Figure 1), a series of transfer events that were almost certainly human-mediated, though the details remain unknown. In Madagascar, plague was most likely transported from its introduction point on the coast to the central highlands, where it became permanently established, via the railroad linking Toamasina and Antananarivo [7]. More recently, plague was most likely reintroduced to Mahajanga via the transport of infected rats and fleas together with foodstuffs from the central highlands. Indeed, our data suggest multiple transfers between Mahajanga and the central highlands, all likely human-mediated. Additional long distance transfers of *Y. pestis* in Madagascar are suggested by the multiple subclades identified in cities/communes such as Antananarivo and Andina Firaisana (Figure 3, S1, Table S1).

Though long distance transfers of *Y. pestis* undoubtedly occur, it is unclear how often such transfers result in the successful establishment of the transferred genotypes in new locations. At least one transfer to Mahajanga became successfully established and underwent local cycling as evidenced by the Mahajanga I.A subcluster described here (Figure 2A). However, many of the other examples of long distance transfers where multiple subclades were found in a single location are not as clear regarding the establishment of the transferred subclade(s). Antananarivo, for example, is clearly dominated by subclade I.A with only 1–2 representatives of each of the other five subclades identified there (Figure 3, S1, Table S1), suggesting that the presence of these alternative subclades may have been only transitory.

Successful establishment of subclades in new locations following a long distance transfer may be related to adaptive advantages possessed by some genotypes [57]. For instance, subclade I.A appears to be particularly successful in our analysis. The earliest subclade I.A isolate in our dataset was collected in 1974 from the Ambositra district (Table S1), one of the most active plague districts in Madagascar [11]. Subsequent isolates indicate that this subclade continued to exist in a small area of the Ambositra district but also became well established over a large geographic area including and surrounding the capital, Antananarivo. This subclade was also successfully introduced to and established in Mahajanga and appears to have been transferred to the Fianarantsoa district, though it is unclear whether or not it became established there (Figure 3, S1, Table S1). This widespread geographical success may indicate that this subclade possesses an adaptive advantage that enhances its ability to be transferred long distances and become established in new locations [57]. Alternatively, the particular success of this subclade may simply be due to chance.

The central highlands focus remains the most active plague focus in Madagascar [11] and is, consequently, a likely place for new genotypes to emerge. This is particularly true for those central highlands districts with the highest plague activity. For instance, the three unique ribotypes identified in a previous study belonged to isolates from two highly active districts, Ambositra and Ambohimahasoa [24]. Here, isolates belonging to Group II and its subclades were found in three highly active districts, Betafo, Manandriana and Ambositra (Figure 3, S1). As discussed above, Ambositra may also have been the district of origin for the highly successful subclade I.A. Overall, the Ambositra district was one of the two most diverse districts in our analysis, containing representatives from six different subclades (Figure 3, Table S1). This diversity is consistent with the Ambositra district's status as one of the three most important plague districts in Madagascar [8], [11].

The maintenance and spread of *Y. pestis* in Madagascar is a dynamic and highly active process, depending on the natural cycle between the black rat and its flea vectors as well as human activity. *Y. pestis* in Madagascar is maintained in multiple, genetically distinct, geographically separated subpopulations, likely via the black rat. The exact geographic landscape of these subpopulations is probably ever changing, with some subclades going extinct or decreasing in frequency (e.g., subclade I.K), new subclades emerging and becoming established and some subclades being transferred to new locations, where they may become established either temporarily or more long-term. Much of the long distance spread of *Y. pestis* in Madagascar is likely due to human activities that allow for the transport of plague infected rats and fleas from one location to another.

## Supporting Information

Figure S1 **Map of Madagascar.** Districts (gray shaded and labeled A–Y) and cities/communes (numbered points) where *Y. pestis* isolates analyzed in the study were collected are indicated. The capital, Antananarivo, is marked with a star.
(PDF)

Table S1 **Isolates used in this study.**
(XLS)

Table S2 **SNP assay primers and probes.**
(XLS)
