# Supplementary material for: Phylogeography and Molecular Epidemiology of Yersinia pestis in Madagascar
Source: PLoS Negl Trop Dis. 2011 Sep 13;5(9):e1319. doi: 10.1371/journal.pntd.0001319 (PMC3172189; doi:10.1371/journal.pntd.0001319)
Supplement: Figure S1 — Map of Madagascar. Districts (gray shaded and labeled A–Y) and cities/communes (numbered points) where Y. pestis isolates analyzed in the study were collected are indicated. The capital, Antananarivo, is marked with a star. (PDF) [file pntd.0001319.s001.pdf]

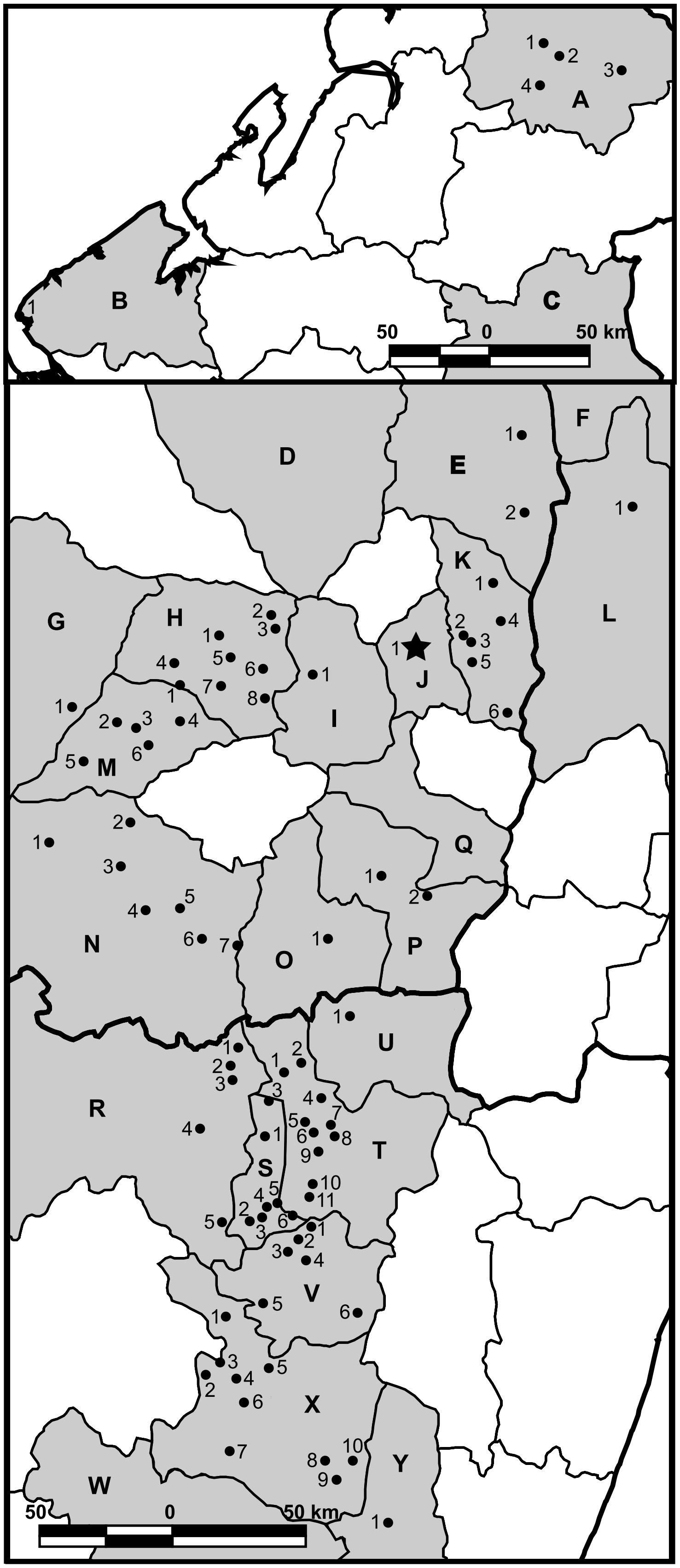

A. Bealanana
1. Beandrarezona
2. Bealanana
3. Marotolana
4. Ambatosia

B. Mahajanga
1. Mahajanga

C. Mandritsara

D. Ankazobe

E. Anjozorobe
1. Betatao
2. Andranokontona

F. Ambatondrazaka

G. Tsiroanomandidy
1. Mahasolo

H. Miarinarivo
1. Ambatomanjaka
2. Soavimbazaha
3. Zoma Bealoka
4. Analavory
5. Miarinarivo
6. Soamahamanina
7. Manazary
8. Mandiavato

I. Arivonimamo
1. Arivonimamo

J. Antananarivo
1. Antananarivo

K. Manjakandriana
1. Ankazondandy
2. Ambanitsena
3. Carion
4. Ranovao
5. Alarobia
6. Merikanjaka

L. Moramanga
1. Amboasary Gara

M. Soavinandriana
1. Ampefy
2. Mahavelona
3. Ampary
4. Soavinandriana
5. Tamponala
6. Amberomanga

N. Betafo
1. Mandoto
2. Fidirana
3. Ankazomiriotra
4. Ambohimanambola
5. Soavina Antanety
6. Mahaiza
7. Tritriva

O. Antsirabe
1. Soanindrariny

P. Antanifotsy
1. Ambatomiady
2. Ambohitompoina

Q. Ambatolampy

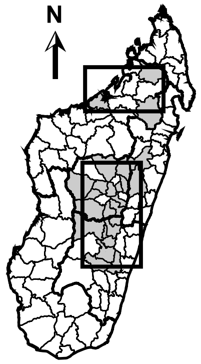

R. Ambatofinandrahana
1. Ambatomifanongoa
2. Ambondromisotra
3. Soavina
4. Ambatofinandrahana
5. Fenoarivo

S. Manandriana
1. Ambatomarina
2. Talata Vohimena
3. Ambohipo
4. Andakatanikely
5. Ambovombe
6. Anjoma Nandihizana

T. Ambositra
1. Mahazina Ambohipierenana
2. Ilaka Afovoany
3. Ihadilanana
4. Tsarasaotra
5. Andina Firaisana
6. Ivony Miaramiasa
7. Ambositra
8. Ankazoambo
9. Ivato
10. Ambalamanakana
11. Ankazomivady

U. Fandriana
1. Tsarazaza

V. Ambohimahasoa
1. Fiadanana
2. Camp Robin
3. Sahatona Tamboharivo
4. Vohiposa
5. Isaka
6. Ambalakindresy

W. Ambalavao

X. Fianarantsoa
1. Fanjakana
2. Mahazoarivo
3. Ambalamidera II
4. Soatanana
5. Nasandratrony
6. Andoharanomaitso
7. Vohimarina
8. Vohitrafeno
9. Vinanitelo
10. Andranomiditra

Y. Ikongo
1. Ikongo
